# The vasculogenic mimicry related signature predicts the prognosis and immunotherapy response in renal clear cell carcinoma

**DOI:** 10.1186/s12885-024-12107-x

**Published:** 2024-04-05

**Authors:** Yuming Gu, Qinqin Huang, Yun Wang, Haixia Wang, Zhenhua Xiang, Yu Xu, Xin Wang, Weiguo Liu, Aiju Wang

**Affiliations:** 1 Department of Traditional Chinese medicine, School of Clinical Medicine, Affiliated Hospital of Weifang Medical University, Weifang Medical University, Weifang, Shandong Province 261042 China; 2 Weifang Medical University, Weifang, Shandong Province 261042 China; 3 Clinical Research Center, Affiliated Hospital of Weifang Medical University, Weifang, Shandong Province 261042 China; 4 Changchun University of Chinese Medicine, Changchun, Jilin Province 130117 China

**Keywords:** Clear cell renal cell carcinoma, Vasculogenic mimicry, Prognosis, Immunotherapy efficacy, Molecular docking, CDH5

## Abstract

**Background:**

Clear cell carcinoma of the kidney is a common urological malignancy characterized by poor patient prognosis and treatment outcomes. Modulation of vasculogenic mimicry in tumor cells alters the tumor microenvironment and the influx of tumor-infiltrating lymphocytes, and the combination of its inducers and immune checkpoint inhibitors plays a synergistic role in enhancing antitumor effects.

**Methods:**

We downloaded the data from renal clear cell carcinoma samples and vasculogenic mimicry-related genes to establish a new vasculogenic mimicry-related index (VMRI) using a machine learning approach. Based on VMRI, patients with renal clear cell carcinoma were divided into high VMRI and low VMRI groups, and patients’ prognosis, clinical features, tumor immune microenvironment, chemotherapeutic response, and immunotherapeutic response were systematically analyzed. Finally, the function of CDH5 was explored in renal clear cell carcinoma cells.

**Results:**

VMRI can be used for prognostic and immunotherapy efficacy prediction in a variety of cancers, which consists of four vasculogenic mimicry-related genes (CDH5, MMP9, MAPK1, and MMP13), is a reliable predictor of survival and grade in patients with clear cell carcinoma of the kidney and has been validated in multiple external datasets. We found that the high VMRI group presented higher levels of immune cell infiltration, which was validated by pathological sections. We performed molecular docking prediction of vasculogenic mimicry core target proteins and identified natural small molecule drugs with the highest affinity for the target protein. Knockdown of CDH5 inhibited the proliferation and migration of renal clear cell carcinoma.

**Conclusions:**

The VMRI identified in this study allows for accurate prognosis assessment of patients with renal clear cell carcinoma and identification of patient populations that will benefit from immunotherapy, providing valuable insights for future precision treatment of patients with renal clear cell carcinoma.

**Supplementary Information:**

The online version contains supplementary material available at 10.1186/s12885-024-12107-x.

## Background

Renal cell carcinoma (RCC) is the commonest and deadliest urologic malignancy. Clear cell renal carcinoma (ccRCC) is the most common histologic subtype of renal cell carcinoma, accounting for 70–75%of all cases [1, 2]. Metastases are present in approximately 50% of patients with localized RCC and less than 14% of these patients have a five-year survival rate [3]. Renal cell carcinoma responds poorly to conventional radiotherapy and chemotherapy, so surgery remains the most effective treatment for RCC [4]. Immune checkpoint inhibitor (ICI)-based immunotherapy has emerged as a promising therapeutic approach for the treatment of various cancers by mediating the regeneration of depleted T cells and thus killing tumor cells. Although ICI therapy is the standard of care for advanced renal cancer, only a small proportion of patients derive long-term and significant clinical benefit from ICI therapy [4, 5]. Therefore, there is an urgent need to develop biomarkers that predict prognosis and better ICI therapy stratification in patients with renal cell carcinoma.

Angiogenesis is an important hallmark of renal clear cell carcinoma and is strongly associated with malignant disease progression and poor treatment outcome in ccRCC cell tumors [6]. Several current treatments for ccRCC primarily target the proliferation of vascular endothelial cells (ECs) within the tumor [7, 8]. However, a large number of preclinical and clinical studies have shown that malignant tumor progression occurs after antiangiogenic therapy, suggesting that there may be another potential mechanism for angiogenesis that bypasses the formation of vascular endothelial cells during tumor progression [9]. Vasculogenic mimicry is closely related to the immune microenvironment in tumor development [10]. Recent studies have shown that angiogenic mimicry (VM), another vasculature system in which tumor cells mediate angiogenesis, plays a key role in the progression of ccRCC, and that targeted blockade of VM in tumors may be a novel anti-angiogenic therapeutic strategy for ccRCC [11, 12].

In this study, we collected vasculogenic mimicry-related genes from PubMed literature and ccRCC patients based on these genes. The vasculogenic mimicry-related index (VMRI) was established based on four vasculogenic mimicry-related genes using a machine learning algorithm. Afterwards, we further evaluated the relationship between VMRI and ccRCC patients’ prognosis, clinical traits, tumor immune microenvironment, immunotherapy and drug treatment efficacy, and the results were comprehensively validated in multiple ways. We also identified natural small molecules with high affinity for CDH5, MMP9, MAPK1 and MMP13 by molecular docking method. Finally, the function of CDH5 in ccRCC was verified in vitro.

## Materials and methods

### Obtaining data from patients with renal clear cell carcinoma and identifying vasculogenic mimicry-related genes

We extracted 42 vasculogenic mimicry-related genes (Table S1) in PubMed publications including review articles [13–17]. Expression profiling data, corresponding clinical information, and pathologic sections of 532 patients with renal clear cell carcinoma were then downloaded from The Cancer Genome Atlas (TCGA) (https://portal.gdc.cancer.gov/) database. Data from the GSE15641, GSE36895, and GSE53757 cohorts were obtained from the Gene Expression Omnibus (GEO) (https://cancergenome.nih.gov/) database as well as clinical data from 91 patients with renal clear cell carcinoma were downloaded as an external validation set from the ICGC database (https://dcc.icgc.org/) [18]. We also downloaded the IMvigor210 immunotherapy data cohort, a set of expression profiling data and corresponding clinical information for a group of patients with uroepithelial cancer treated with an anti-PD-L1antibody (atezolizumab) (http://research-pub.gene.com/IMvigor210CoreBiologies) [19].

### Construction and validation of a vasculogenic mimicry related index and histological validation at the protein level

The cox proportional-hazards model a semiparametric regression model that simultaneously examines the relationship between multiple risk factors and the occurrence and timing of event outcomes, thereby overcoming the shortcomings of the single-factor limitation in simple survival analysis.

We first collected vasculogenic mimicry-related genes, followed by differential expression and prognostic analyses between renal clear cell carcinoma tumor tissues and normal tissues, and finally the vasculogenic mimicry-related index (VMRI) was derived using multifactorial cox regression analysis. The VMRI of each renal clear cell carcinoma patient was obtained according to the following formula: vasculogenic mimicry related index (VMRI) = Coef(Gene1) × Expr(Gene1) + Coef(Gene2) × Expr(Gene2) +… + Coef(Genen) × Expr(Genen), in which Expr(Genen) represents the expression of a particular gene and Coef(Genen) is the coefficient obtained from multifactor Cox analysis of genes. GSE15641, GSE36895, GSE53757 and ICGC cohorts were subjected to elimination of batch effects and combined as an external validation cohort. We further searched the CPTAC database (https://pdc.cancer.gov/pdc/) and the Human Protein Atlas database (https://www.proteinatlas.org/) [20] and obtained the results of CDH5, MMP9, MAPK1 and MMP13 expression levels at the protein level and verified by immunohistochemical staining smears.

### Gene set enrichment analysis (GSEA) and immune infiltration analysis

We downloaded the enriched pathway gene sets from the MSigDb database (https://www.gsea-msigdb.org/gsea/msigdb/). The R package “clusterProfiler” was used to evaluate the major enriched pathways in the Cluster 1 (C1), Cluster 2 (C2) and high VMRI groups, and the HALLMARK, c5GO and c2KEGG gene sets were set as the enriched gene sets, and the filtering conditions were|NES| > 1, nominal *p*-value < 0.05. value < 0.05. Tumor purity, stromal score, immune score and ESTIMATE score were calculated for each renal clear cell carcinoma patient using the “ESTIMATE” package in the R program [21]. We used the Single Sample Gene Set Enrichment Analysis (ssGSEA) algorithm to study the immune microenvironment analysis based on different immune cell types between the high/low VMRI groups. Additionally XCELL and CIBERSORT software were used to analyze the association of VMRI with the level of immune cell infiltration. Pathology section lymphocyte scores were graded and described tumor inflammation using a semi-quantitative scoring system (0–5). 0–1 was classified as low immune cell infiltration, 2–3 as moderate immune cell infiltration, and 4–5 as high immune cell infiltration.

### Analysis of immunotherapy response

We obtained Tumor Immune Dysfunction and Rejection (TIDE) algorithm scores for pan-cancer from the TIDE database (http://tide.dfci.harvard.edu/) [22] to assess the response to immunotherapy in patients with clear cell carcinoma of the kidney. High TIDE scores tend to be associated with poorer response to immunotherapy and a higher likelihood of immune escape. In addition, we performed VMRI constructs on pan-cancer patients thereby predicting the predictive value of VMRI for immunotherapy in pan-cancer. We further downloaded the IPS scoring (Immunophenotype score, the higher the score the better the therapeutic response to immune checkpoint (PD-1 and CTLA4) inhibitors) in renal clear cell carcinoma from the TCIA database (https://tcia.at/home) [23], and in turn analyzed its association with VMRI, and these results were used to assess the predictive value of VMRI for the therapeutic effects of common immune checkpoints. Finally, we also extracted clinical information from the IMvigor210 dataset of 348 patients with uroepithelial carcinoma who received anti-PD-L1 immunotherapy (atezolizumab) to assess the difference in high/low VMRI between the anti-PD-L1 immunotherapy groups (responding or non-responding) [19].

### Molecular docking simulation

Molecular docking is a bioinformatics-based simulation method that evaluates interactions between molecules and predicts their binding modes and affinities using a computerized platform. We used Schrödinger software to screen small molecule drugs that bind to target target proteins and performed molecular docking simulations. We downloaded the protein structures of the target targets (CDH5-3PPE, MMP9-5UE4, MAPK1-7NQQ, and MMP13-5UWL) from the PDB database, and collected 11,335 natural small molecule drugs from the PubChem database (https://pubchem.ncbi.nlm.nih.gov/). We set Use PROPKA pH to 7.0 and energy minimization of protein structures, Precision to standard precision, and simulated the binding poses of CDH5, MMP9, MAPK1, and MMP13 with small molecule drugs by the Glide module in Schrödinger software.

### Cell culture and small interfering RNA (siRNA) transfection sequences

We purchased and used the human renal clear cell carcinoma cell lines 769-P and 786-O from the Shanghai Cell Bank of the Chinese Academy of Sciences. 769-P and 786-O cells were cultured in adherent wall culture in medium containing 5% fetal bovine serum. The cells were cultured at 37 °C with 5% carbon dioxide.

We designed siRNA for CDH5 to silence CDH5 expression in human renal clear cell carcinoma cell lines 769-P and 786-O cells to investigate the role of CDH5 in renal clear cell carcinoma. Inoculate 769-P and 786-O cells in each well of a 6-well plate and put 2.5 ml of antibiotic-free growth medium into each well. When the cell fusion reached about 70%, the above medium was removed, 1.5 ml of fresh serum-free growth medium was added, and siRNA and lipo3000 RNAi mix was prepared. Add 100 pmole of siRNA to 250 µL of serum-free growth medium and mix gently. lipo3000 RNAi was mixed before use, and 5 µL of lipo3000 RNAi was added to 250 µL of growth medium for dilution and incubated for 5 min at room temperature; mix the diluted siRNA and lipo3000 RNAi and incubate for 20 min at room temperature. Add the mixture to 6-well plates inoculated with 769-P and 786-O cells, change the medium containing serum and incubate the cells for 48 h to analyze the effect of siRNA silencing. The siRNA targeting CDH5 was purchased from Sigma. qPCR was performed at the mRNA level to detect the silencing effect of CDH5. RNA extraction was performed after sample collection to remove impurities from the sample, sample lysis, purification and removal of DNA, proteins and other impurities, pre-denaturation: 95℃, 5 min; cycling reaction: 95℃, 30s; 55℃, 30s; 72℃, 30s; 40 cycles. The primers used for qPCR analysis were as follows: CDH5 forward:5′-TCACCTTCTGCGAGGATATGG-3′, reverse: 5′- GAGTTGAGCACCGACACATC-3′.

### Cell counting kit-8 (CCK8) cell activity assay and plate cloning assay

CCK8 and plate cloning were used to determine the cell proliferation ability. In the CCK8 assay, take the cells in logarithmic growth state and formulate to add about 2 × 10³ cells/100ul per well, 100 ul per well, into a 96-well cell culture plate. Add 10 ul of CCK-8 solution to the 96-well cell culture plate and incubate at 37 °C for 0.5-4 h. Absorbance was detected using a single wavelength of 450 nm. For plate cloning experiments, cells in the logarithmic growth phase were trypsin digested and resuspended in complete medium (basal medium + 10% fetal bovine serum) into cell suspension and counting. Each experimental group was inoculated in 6-well plate culture plates, and the incubation was continued up to 14 days, with fluid change and observation of cell status every 3 days in the middle. After cloning completion, the cells were photographed under a microscope and then washed once with PBS. Each well was fixed by adding 1 mL of 4% paraformaldehyde for 30–60 min and washed once with PBS. The images were detected using a fluorescence microscope. Each experiment was repeated three times.

### Cell migration capacity assay

Transwell and Wound-healing experiments were performed to determine the cell invasion capacity. For Transwell experiments, 769-P and 786-O cells were first starved for 24 h to remove the effect of serum. Cells with 80% confluence were taken for digestion and centrifuged, and the cells were resuspended. FBS medium was added to the lower chamber of the 24-well plate. Then the small chamber of Transwell was placed in the 24-well plate with forceps, and cell suspension was added to the upper chamber of Transwell in each well, and fixed staining was performed after 24 h of incubation. The cells were stained with crystal violet for 10 min, and after appropriate air-drying, the cells were observed and counted in selected fields under the microscope. For wound-healing experiments, a marker pen was used to draw horizontal lines evenly, about every 1 cm, across the wells on the back of a 6-well plate with a straightedge. The 769-P and 786-O cells were added to the wells, and the next day, the lines were drawn with the tip of the gun compared to the straightedge. The cells were washed three times with PBS and then cultured in a 37 °C, 5% CO2 incubator. Samples were taken at 48 h using an inverted microscope to observe the migration of cells at a specific location and photographed. Each experiment was repeated three times.

### The Kaplan-Meier method

The Kaplan-Meier curve, also known as the survival curve, is a commonly used method of survival analysis, which analyzes the effect of a single factor on survival, and is used to estimate the survival rate of a patient and to draw survival curves. The survival curve is a continuous step curve drawn with survival time as the horizontal axis and survival rate as the vertical axis to illustrate the relationship between survival time and survival rate.

### Statistical analysis

The Wilcoxon and Kruskal-Wallis tests were employed to compare two or more groups. *p*-values ≤ 0.05 were considered statistically significant. All statistical analyses were performed by R. Image J and GraphPad software were used for experimental data processing and plotting.

## Results

### Identification of vasculogenic mimicry-related subtypes in renal clear cell carcinoma

**Fig. 1:**
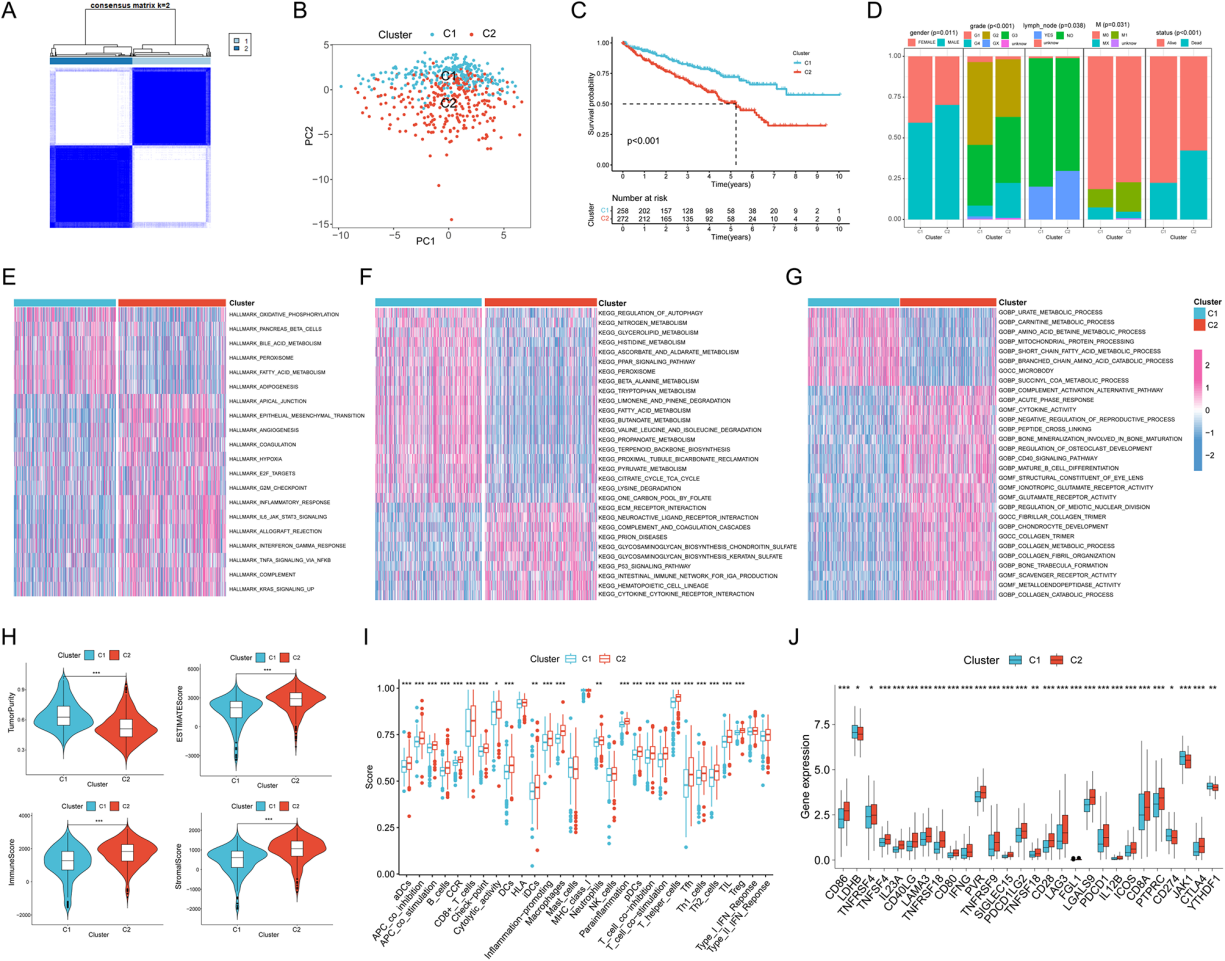
Identification of vasculogenic mimicry-related isoforms. (**A**) Clustering heat map of ccRCC divided into two clusters. PCA analysis (**B**), survival curves (**C**) and clinical shapes (**D**) were analyzed between the two clusters. Heatmaps of enrichment in HALLMARK pathway (**E**), KEGG (**F**) and GO (**G**) pathway enrichment between the two clusters were identified by enrichment analysis. (**H**) Tumor purity, ESITIMATE score, immune score and stroma score between the two clusters. (**I**) Comparison of differences in immune function and immune cell infiltration levels between the two clusters by ssGSEA. (J) Analysis of common immune checkpoint differences between the two clusters. Note * *p* < 0.05, **p < 0.01, ***p < 0.001

We analyzed renal clear cell carcinoma patients based on 42 vascular survival mimicry-related genes collected from PubMed. The results showed that ccRCC patients could be well divided into two clusters, which both had good internal consistency and stability (Fig. 1A, Figure S1). PCA analysis showed that renal clear cell carcinoma patients could be well divided into two clusters (Fig. 1B). The survival curve results showed that Cluster2 had a poorer prognosis compared with Cluster1 (Fig. 1C). In clinical characterization, the two clusters differed in gender, grading, presence of lymph node metastasis, M (distant organ metastasis) and patient survival (*P* < 0.05), and significantly in tumor grading and survival (*P* < 0.001). Cluster 2 had a significantly higher proportion of males, high grading, presence of lymph node metastasis, M1 (metastasis to distant organs) and death compared to patients with renal clear cell carcinoma in Cluster 1 (Fig. 1D). We then investigated the enriched pathways between the two clusters by gene set enrichment analysis, which showed that Cluster2 was enriched in the pathways Hypoxia, Inflammatory response, Ecm receptor interaction and Cytokine activity (Fig. 1E-G).

We further analyzed the differences in the tumor immune microenvironment between the two clusters. Firstly, the results by ESITIMATE algorithm showed that Cluster2 patients had lower tumor purity and higher ESITIMATE scores, immune scores and stromal scores compared to Cluster1 (Fig. 1H). Subsequent analysis by ssGSEA revealed that most of the immune cell infiltration levels and immune functions were significantly higher in Cluster2 patients than in Cluster1 patients (Fig. 1I). We further analyzed common immune checkpoints and found that the expression levels of most immune checkpoints were significantly higher in Cluster2 patients than in Cluster1 patients (Fig. 1J).

### Vasculogenic mimicry related index and columnar plot construction

**Fig. 2:**
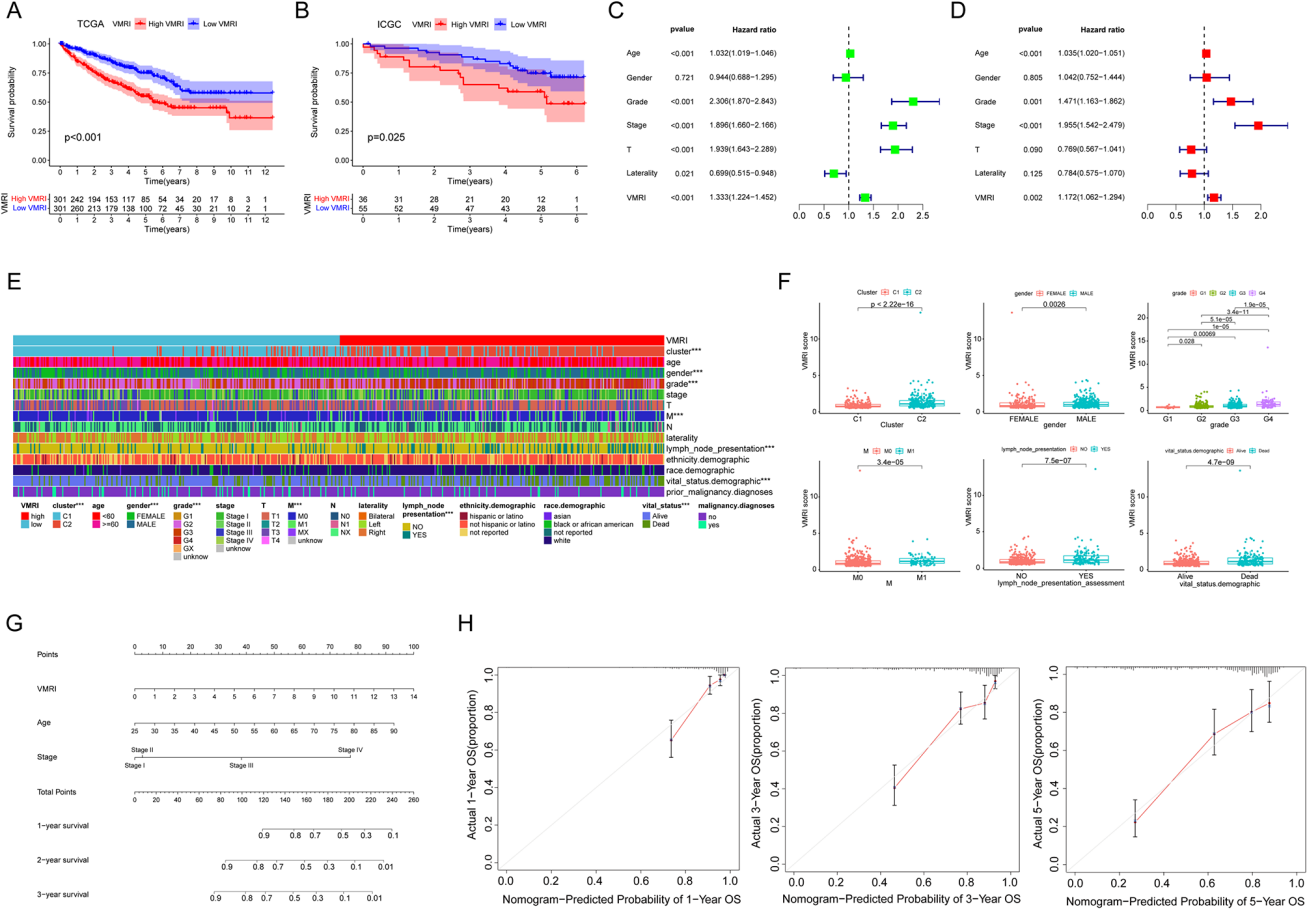
Vasculogenic mimicry related index predicts prognosis in patients with renal clear cell carcinoma. (**A**) Survival curve of high/low VMRI group in TCGA cohort. (**B**) Survival curves of high/low VMRI group in ICGC cohort. (**C**, **D**) Univariate and multivariate regression analysis. (**E**) Differences in clinical traits between high/low VMRI groups. (**F**) Differences in VMRI between clinical traits. (**G**) Column line graphs based on age, staging, and VMRI. (**H**) Calibration curves for 1-, 3-, and 5-year overall survival. Note * *p* < 0.05, **p < 0.01, ***p < 0.001

After differential and prognostic screening of VM genes, we identified four VM-related genes with independent prognostic value by multifactorial Cox regression analysis and thus constructed with vasculogenic mimicry-related VMRI. The coefficients of the four genes for the construction of the VMRI are shown in Table S2. We analyzed the survival status of the patients with high/low VMRI in the TCGA cohort by means of the KM survival curve. The results showed that patients in the high VMRI group had a worse prognosis compared to the low VMRI group (Fig. 2A). We also externally validated the above results by collecting survival information of ccRCC patients through the ICGC database, and in the consistency, patients in the high VMRI group had a worse prognosis (Fig. 2B). The results of univariate and multivariate regression analyses showed that age, grade, stage and VMRI were independent risk factors for ccRCC patients (Fig. 2C-D). To further validate the clinical significance of VMRI, we analyzed the associations between VMRI and different clinical traits, and found that VMRI was significantly associated with clustering, gender, grading, M, the presence of lymph node enlargement, and patient status (*p* < 0.05) (Fig. 2E). We also compared the differences in VMRI between the different clinical traits mentioned above, and showed that VMRI was higher in patients with Cluster2, male, high grade, M1, lymph node enlargement, and deceased, suggesting that the higher the patient’s VMRI, the more advanced the tumor was (Fig. 2F). We constructed nomograph for predicting the prognosis of patients with ccRCC based on age, stage, and VMRI of patients with renal clear cell carcinoma (Fig. 2G). The calibration curves showed that the 1-, 3- and 5-year column line plots exhibited good predictive accuracy compared with the reference line (Fig. 2H). These results suggest that VMRI can accurately and reliably predict the survival outcome of patients with renal clear cell carcinoma.

### Vasculogenic mimicry-related index predicts pan-cancer prognosis

**Fig. 3:**
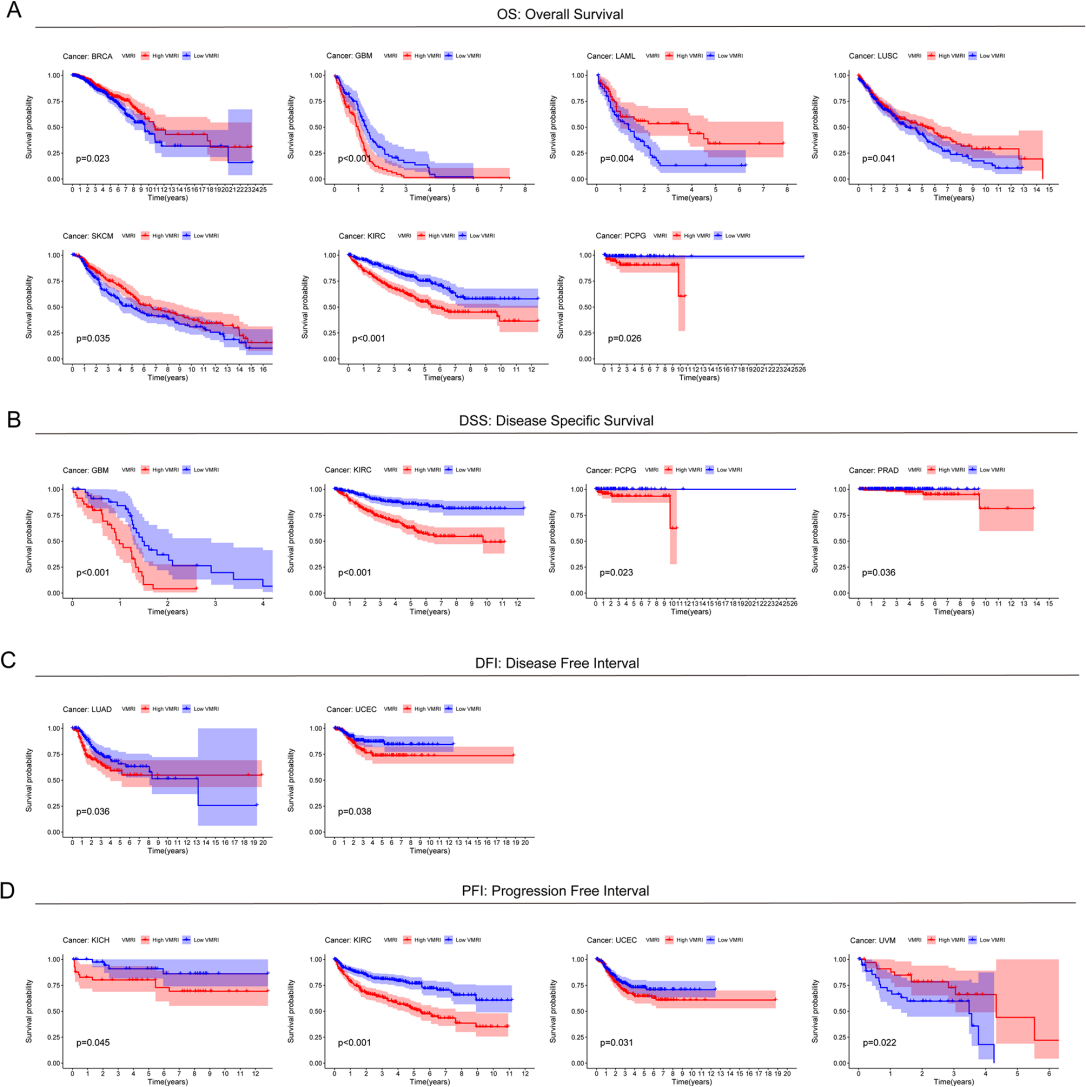
Predictive value of VMRI in other cancers. (**A**) OS survival curves of patients in the high/low VMRI group in GBM, KIRC, PCPG, BRCA, LAML, LUSC and SKCM. (**B**) DSS survival curves of patients in the high/low VMRI group in GBM, KIRC, PCPG and PRAD. (**C**) DFI survival curves of patients in the high/low VMRI group in LUAD and UCEC. (**D**) PFI survival curves of patients in the high/low VMRI group in KICH, KIRC, UCEC, and UVM

To further investigate the value of VMRI in prognostic prediction of other cancer patients, we used the above modeling formula for VMRI to calculate VMRI in other cancer patients and plotted the KM survival curves for high/low VMRI groups. For Overall Survival (OS), patientsin the high VMRI group had a poorer prognosis in GBM, KIRC, and PCPG, while patients in the low VMRI group had a poorer prognosis in BRCA, LAML, LUSC, and SKCM (Fig. 3A). For Disease Specific Survival (DSS), patients in the high VMRI group had a poor prognosis compared to patients in the low CMRI group in GBM, KIRC, PCPG and PRAD (Fig. 3B). For Disease Free Interval (DFI), patients in LUAD and UCEC had a poor prognosis in the high VMRI group compared to patients in the low VMRI group (Fig. 3C). For Progression Free Interval (PFI), patients in the high VMRI group had a poor prognosis compared with patients in the low CMRI group in KICH, KIRC and UCEC. And in UVM, patients in the low VMRI group had a poor prognosis (Fig. 3D).

### A study of the correlation between VMRI and the tumor microenvironment

**Fig. 4:**
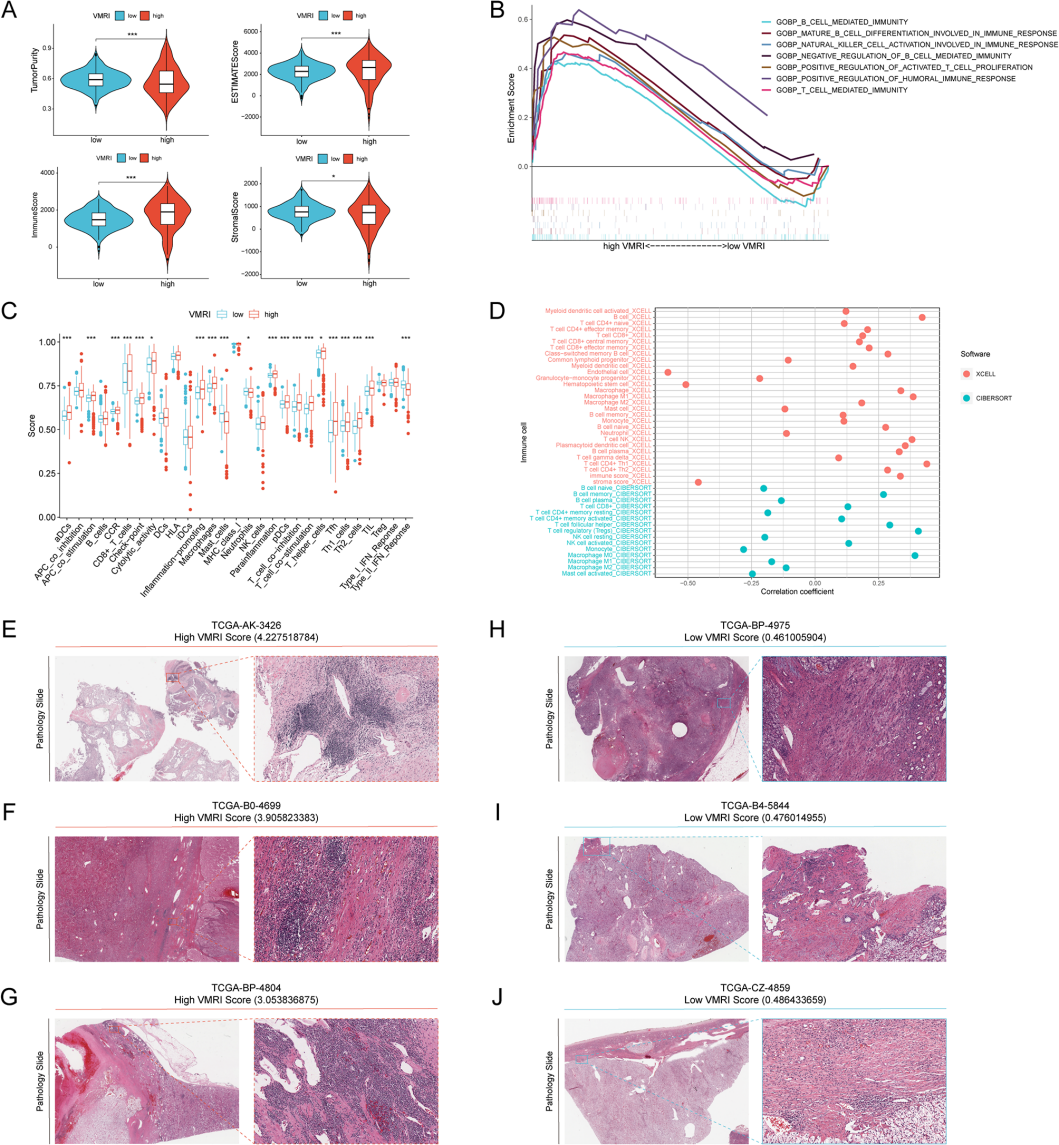
Correlation analysis between VMRI and tumor microenvironment. (**A**) Comparison of tumor purity, ESTIMATE score, immunity score and stroma score of patients in high/low VMRI group. (**B**) GSEA analysis of patients in the high VMRI group. (**C**) Comparison of immune cell infiltration levels and immune-related functions in patients in the high/low PMGI group by ssGSEA analysis. (**D**) XCELL and CIBERSORT software to analyze the correlation between VMRI and immune cell infiltration. immune infiltration levels of patients in the high VMRI group (**E**-**G**) and patients in the low VMRI group (**H**-**J**) in TCGA pathology sections. Note * *p* < 0.05, **p < 0.01, ***p < 0.001

The ESITIMATE algorithm revealed that patients in the high VMRI group had lower tumor purity and higher ESITIMATE scores, immune scores, and stromal scores compared with patients in the low VMRI group (Fig. 4A).GSEA enrichment analysis showed that patients in the high VMRI group had higher B_CELL_MEDIATED_IMMUNITY, T_CELL_ MEDIATED_IMMUNITY and NATURAL_KILLER_CELL_ACTIVATION_IN_IMMUNE_RESPONSE were significantly enriched (Fig. 4B). ssGSEA analysis showed that compared to the low VMRI group, patients in the high VMRI group had higher levels of immune cell infiltration and immune-related functions and pathways (Fig. 4C). We also found a correlation between VMRI and different immune cell infiltration by XCELL and CIBERSORT software (Fig. 4D). All of these results indicated that patients in the high VMRI group had higher levels of immune infiltration than those in the low VMRI group.

We verified the accuracy of the above results by means of pathological sections of patients with renal clear cell carcinoma, and we found that high levels of immune cell infiltration were present in the tissues of patients in the high VMRI group (TCGA-AK-3426, TCGA-B0-4699, and TCGA-BP-4804) (Fig. 4E-G). Whereas, the level of immune cell infiltration was low in patients in the low CMRI group (TCGA-BP-4975, TCGA-B4-5844 and TCGA-CZ-4859) (Fig. 4H-J).

### Association of VMRI with immunotherapy efficacy

**Fig. 5:**
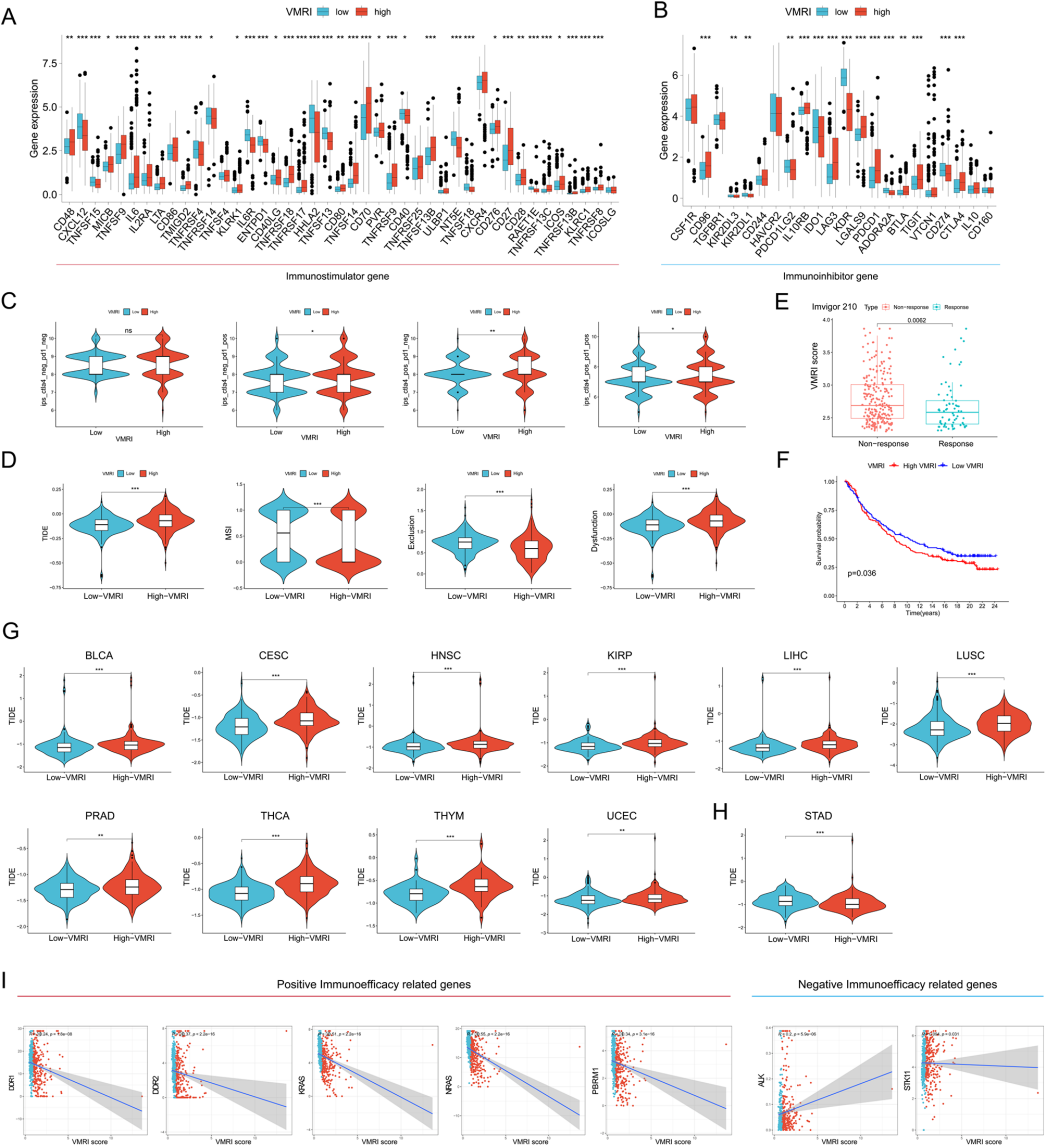
Low VMRI is associated with better immunotherapy response. Differences in immunostimulatory genes (**A**) and immunosuppressive genes (**B**) between high and low VMRI groups. (**C**) IPS scores of anti-PD1(-)CTLA4(-), anti-PD1(+)CTLA4(-), anti-PD1(-)CTLA4(+), and anti-PD1(+)CTLA4(+) blockers in the high and low VMRI groups. (**D**) Differences in TIDE, MSI, Exclusion, and Dysfunction between high and low VMRI groups. (**E**) Differences in VMRI scores between immunotherapy-responsive and non-responsive groups in the Imvigor210 cohort. (**F**) Survival curves for high/low VMRI groups in the Imvigor210 cohort. (**G**-**H**) VMRI score for immunotherapy efficacy in pan-cancer. (**I**) Association of VMRI with genes related to immunotherapy efficacy. Note * *p* < 0.05, **p < 0.01, ***p < 0.001

We first analyzed the differences in common immune checkpoints, including immunostimulatory and immunosuppressive genes, between high/low VMRI subgroups, which resulted in most of the immune checkpoints being significantly overexpressed in the high VMRI group, such as PDCD1 and CTLA4; however, CD274 was highly overexpressed in the low VMRI group (Fig. 5A-B). We then assessed immunogenicity by immunophenotypic core (IPS) scoring to predict patient response to immune checkpoint blockade (anti-PD1 and/or anti-CTLA4), with higher IPS scores indicating better predicted immunotherapy outcomes. We found that anti-PD1, anti-CTLA4 and anti-PD1-CTLA4 combination therapy was more effective in the low VMRI group (Fig. 5C). Lower TIDE scores in patients were associated with a greater likelihood of benefit from immunotherapy and longer survival [22]. Patients in the low VMRI group had lower TIDE scores and Dysfunction and higher Microsatellite instability (MSI) and Exclusion compared to patients in the high VMRI group, suggesting better efficacy of immune checkpoint blockade therapy in this group (Fig. 5D). Subsequent validation of the VMRI for predicting immunotherapy efficacy by external immunotherapy datasets showed that patients in the immunotherapy-responsive group in the Imvigor210 (anti-PD-L1) cohort had significantly lower VMRI values than patients in the non-responsive group, and that patients in the low VMRI group had a higher prognosis (Fig. 5E-F). We also applied the VMRI score to a pan-cancer study to evaluate its value in predicting immunotherapy outcomes in other cancers. It was found that VMRI was able to predict immunotherapy outcomes not only in renal clear cell carcinoma, but also in a wide range of cancers. For example, in BLCA, CESC, HNSC, KIRP, LIHC, LUSC, PRAD, THCA, THYM, and UCEC, patients in the low VMRI group may have higher immunotherapy outcomes, but in STAD, patients in the high VMRI group may have higher immunotherapy outcomes (Fig. 5G-H). Finally, we analyzed the association between immuno-efficacy-related genes and with PCDI and showed that VMRI was significantly negatively associated with positive immuno-efficacy-related genes (DDR1, DDR2, KRAS, NRAS, and PBRM1), while it was significantly positively associated with negative immuno-efficacy-related genes (ALK and STK11) (Fig. 5I). All these results indicated that VMRI could better predict the immunotherapy effect and the low VMRI group responded better to immunotherapy.

### Small molecule drug candidate prediction for core target proteins

**Fig. 6:**
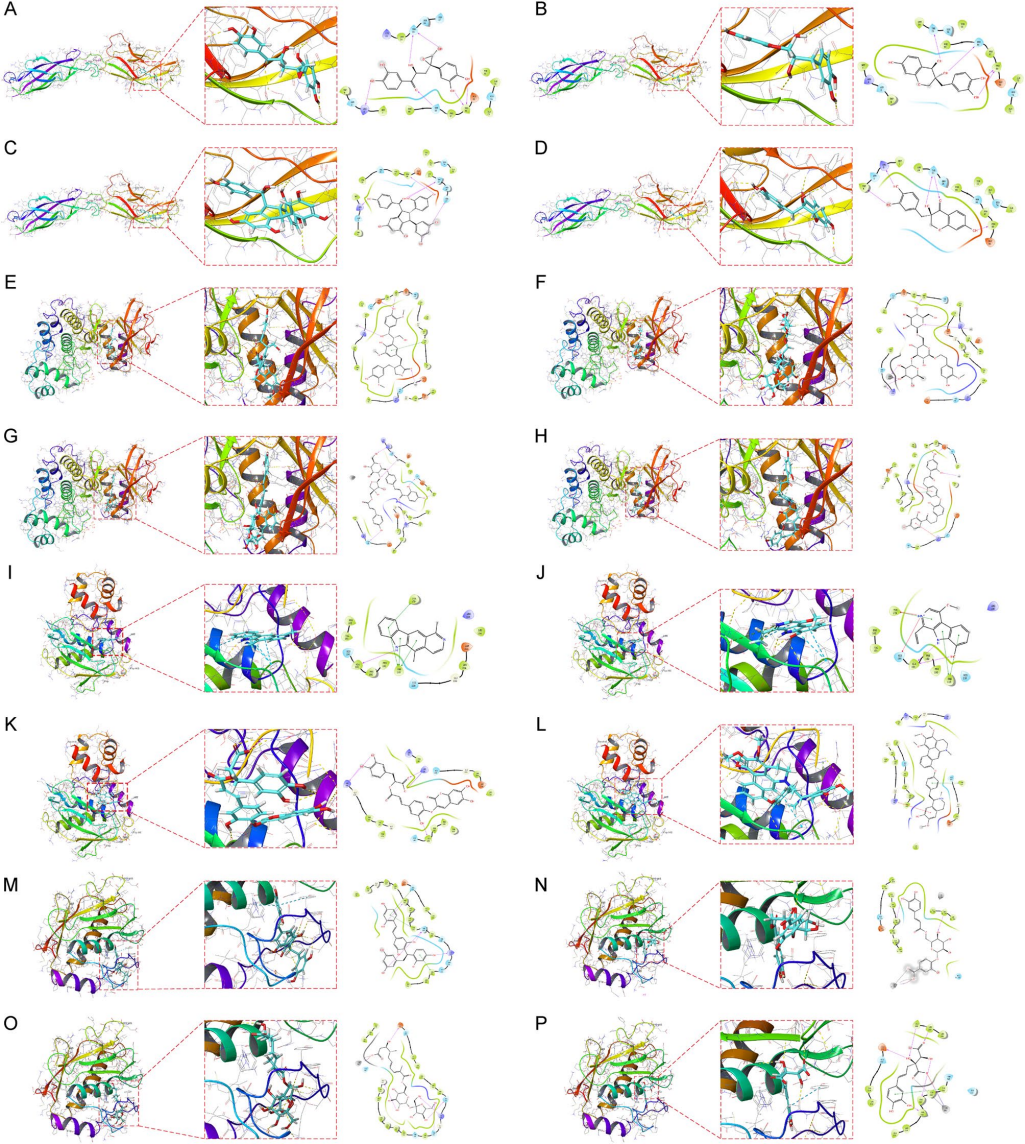
Molecular docking pose. Screening of candidate small molecules for target proteins using molecular docking. The figure shows the docking poses of the CDH5 active pocket with Crassifogenin C (**A**), Episappanol (**B**), Gnetuhainin D (**C**), and Sappanone B (**D**). the MAPK1 active pocket with Isolappaol C (**E**), Leonoside F (**F**), Licorice Glycoside B (**G**), and Littorachalcone (**H**) in docking poses.MMP9 active pocket with Olivacine (**I**), Picrasidine I (**J**), Sagecoumarin (**K**), and Thalifaretine (**L**) in docking poses.MMP13 active pocket with Andalasin A (**M**), Dracunculifoside B (**N**), Glansreginin B (**O**) and Monocaffeyltartaric Acid (**P**) in docking poses

We obtained protein structures of CDH5, MAPK1, MMP9, and MMP13 from the PDB database for molecular docking with natural small molecule compounds. The former four small molecules with the highest binding affinity to the CDH5 binding pocket (Crassifogenin C, Episappanol, Gnetuhainin D and Sappanone B) (Fig. 6A-D). The former four small molecules with the highest binding affinity to the MAPK1 binding pocket (Isolappaol C, Leonoside F, Licorice Glycoside B and Littorachalcone) (Fig. 6E-H). The former four small molecules with the highest binding potency to the MMP9 binding pocket (Olivacine, Picrasidine I, Sagecoumarin, and Thalifaretine) (Fig. 6I-L). The former four small molecules (Andalasin A, Dracunculifoside B, Glansreginin B and Monocaffeyltartaric Acid) with the highest binding potency to the MMP13 binding pocket (Fig. 6M-P). We have also collected and organized links to web sites corresponding to specific information on the above small molecule compounds (Table S3). For example Crassifogenin C forms hydrogen bonds with CDH5 amino acid residues Arg-6, Ser-88, Pro86 and Thr-25, where Arg-6, Pro86 and Thr-25 act as hydrogen bond acceptors and Ser-88 acts as a hydrogen bond donor.Sagecoumarin forms hydrogen bonds with MMP9 amino acid residues Arg-56, Arg- 106 and Ala-191 to form hydrogen bonds, wherein Arg-56, Arg-106 and Ala-191 act as hydrogen bond acceptors.

### Constructing VMRI gene expression and immunohistochemistry

**Fig. 7:**
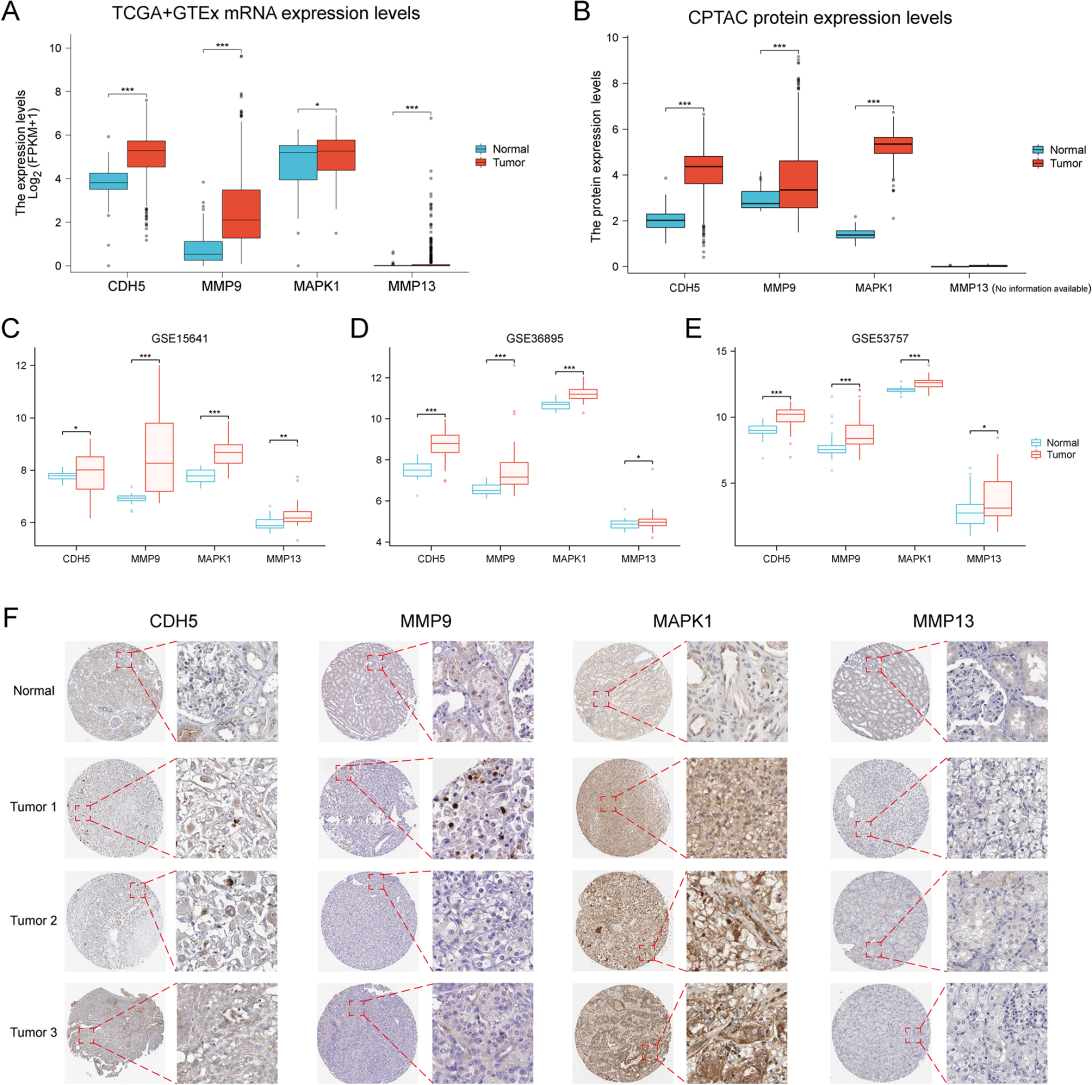
CDH5, MMP9, MAPK1 and MMP13 expression and immunohistochemistry. Differences in the expression levels of CDH5, MMP9, MAPK1 and MMP13 in TCGA and GTEx combined data (**A**), CPTAC (**B**), GSE15641 (**C**), GSE36895 (**D**) and GSE53757 (**E**). (**F**) Immunohistochemical staining of CDH5, MMP9, MAPK1 and MMP13 in renal normal tissues and renal clear cell carcinoma. Note * *p* < 0.05, **p < 0.01, ***p < 0.001

We first analyzed the mRNA expression levels of the four genes (CDH5, MMP9, MAPK1, and MMP13) constructing VMRI in normal kidney tissues and ccRCC by TCGA and GTEx combined data. The results showed that the mRNA expression levels of the above four genes were significantly higher in tumor tissues than in normal tissues (Fig. 7A). After that, we analyzed the protein expression levels of CDH5, MMP9 and MAPK1 in normal kidney tissues and ccRCC by CPTAC database, MMP13 for included in CPTAC database, and the protein expression levels of CDH5, MMP9 and MAPK1 were significantly higher than that in normal tissues in tumor tissues (Fig. 7B). We validated the above mRNA expression levels by GEO external datasets (GSE15641, GSE36895 and GSE53757) and the results were consistent i.e., the mRNA expression levels of CDH5, MMP9, MAPK1 and MMP13 were significantly higher in tumor tissues than in normal tissues (Fig. 7C-E). Finally, we also obtained immunohistochemical staining results of CDH5, MMP9, MAPK1 and MMP13 in renal normal tissues and renal clear cell carcinoma to verify the above results (Fig. 7F).

### Knockdown of CDH5 inhibits the proliferation and migration of renal clear cell carcinoma cells

**Fig. 8:**
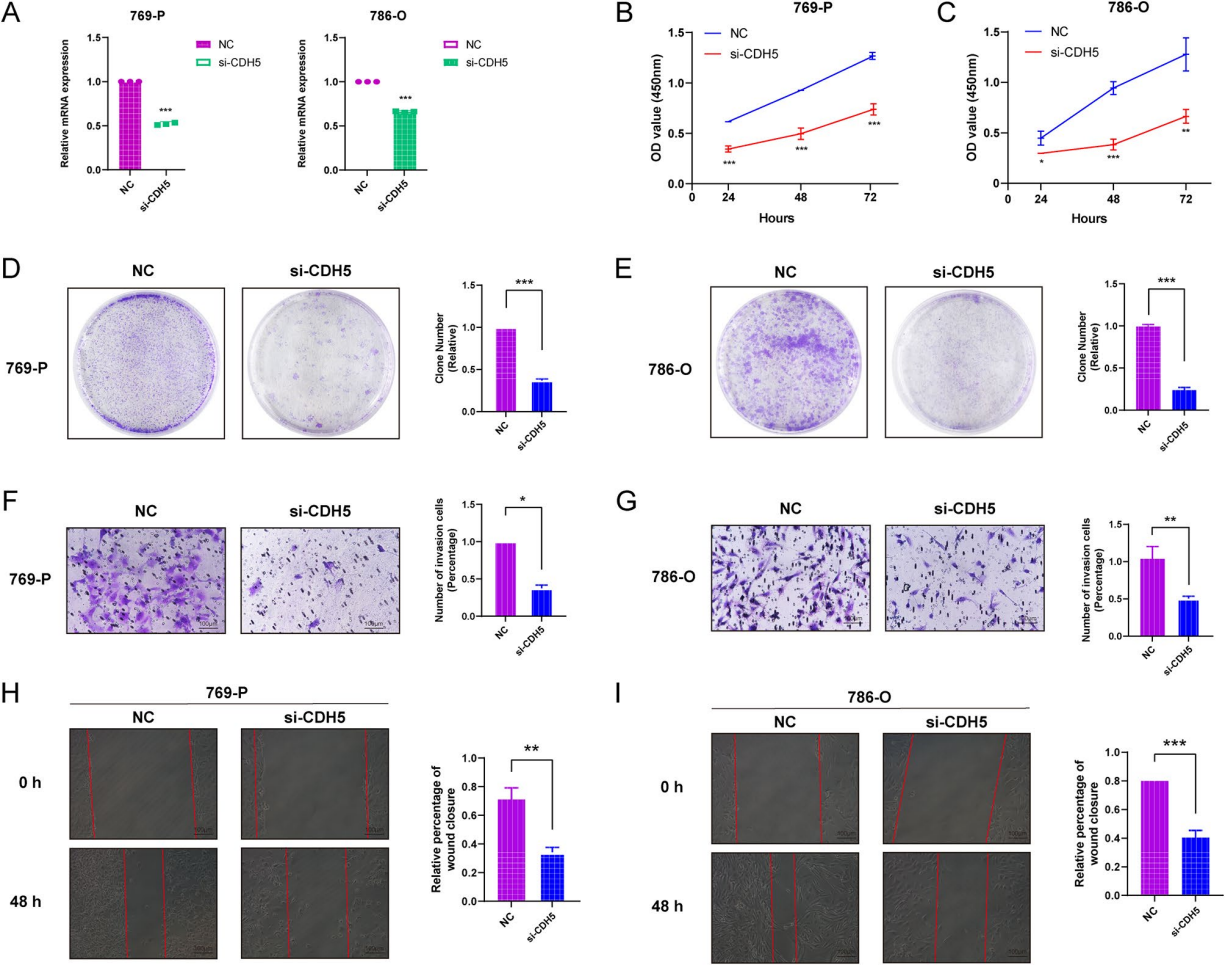
Knockdown of CDH5 inhibits the proliferation and migration ability of renal clear cell carcinoma cells. (**A**) QPCR verified the mRNA expression level of CDH5 in 769-P and 786-O cells after transfection with siRNA. CCK8 viability assay (**B**-**C**), plate cloning assay (**D**-**E**), transwell cell migration ability (**F**-**G**) and Wound-healing assay (**H**-**I**) in 769-P and 786-O cells after transfection with si-CDH5. Note * *p* < 0.05, **p < 0.01, ***p < 0.001

The silencing effect of CDH5 was detected by QPCR, and si-CDH5 could effectively knock down the expression of CDH5 (Fig. 8A). CCK8, plate cloning, Transwell and Wound-healing experiments were performed on 769-P and 786-O cells transfected with si-CDH5, respectively. the results of CCK8 experiments showed that the proliferation ability of 769-P and 786-O cells in the si-CDH5 group was significantly lower than that in the NC group at 24, 48, and 72 h (Fig. 8B-C). The results of plate cloning assay also indicated that the proliferation ability of 769-P and 786-O cells after knockdown of CDH5 was significantly lower than that of NC group (Fig. 8D-E).Transwell assay showed that the cell migration ability of 769-P and 786-O cells was significantly reduced after knockdown of CDH5 (Fig. 8F-G).Wound-healing assay showed that at 48 h later, the migration ability of 769-P and 786-O cells in the si-CDH5 group was significantly lower than that in the NC group (Fig. 8H-I).

## Discussion

Many studies have shown that targeting tumor angiogenesis is an important anti-cancer therapeutic strategy [17, 24–26]. Highly invasive cancer cells can meet their own energy demands and thus form vascular-like channels through deformation and extracellular matrix remodeling, a process known as vasculogenic mimicry. Therefore, exploring the mechanism and function of vasculogenic mimicry will provide some important insights for future cancer therapy [27–29]. The ability of a tumor to occur and develop is closely related to the fact that it can alter the tumor microenvironment in which it resides to help it evade immune surveillance [30]. In order to clarify the relationship between VMRI and the tumor microenvironment of renal clear cell carcinoma. We found that VMRI could differentiate the sensitivity of renal clear cell carcinoma to common chemotherapeutic agents and targeted drugs. The VMRI can assess the efficacy of immunotherapy and chemotherapeutic drugs in ccRCC patients, which can provide a great help for the future treatment of patients with renal clear cell carcinoma.

Vasculogenic mimicry related index (VMRI) is composed of four vasculogenic mimicry related genes including CDH5, MMP9, MAPK1, and MMP13. A current study demonstrated that Cadherin (CDH5) is expressed in glioblastoma cells and induces vasculogenic mimicry under hypoxic conditions [31]. Matrix metalloprotein 9 (MMP9) and matrix metalloprotein 13 (MMP13) belong to the family of metalloproteinases that degrade the extracellular matrix.MMP9 and MMP13 have been reported to play crucial roles in the regulation of tumor vasculogenic mimicry [32–34].Mitogen- Activated Protein Kinase 1 (MAPK1) is a key signaling hub that integrates extracellular signals and plays an important regulatory role in tumor cell proliferation, differentiation, senescence, and drug resistance, and is of wide interest in a variety of diseases [35, 36].

As another application of VMRI efficacy prediction, we demonstrated the feasibility of a structure-based approach to find candidate small molecule drugs that can target core proteins. Among the top four small molecule drugs with the highest affinity for CDH5, Sappanone B, a 3-Benzylchroman derivatives, has been shown to synergize with aminoglycosides in its killing effect on methicillin-resistant Staphylococcus aureus [37]. Littorachalcone with high affinity for MAPK1 is derived from Verbena littoralis and can be used to enhance neuronal synapse growth [38]. Olivacine has the highest affinity for the MMP9 docking pocket, and Olivacine-mediated lysosomal cytosol sputum plays a key role in drug resistance in cancer cells [39]. Although the specific mechanisms of these candidate small molecule compounds have yet to be explored in depth, our findings suggest that they have great potential in vasculogenic mimicry in renal clear cell carcinoma.

There are some limitations to this study; first, the data for our analysis were obtained from public databases, which may have led to some case selection bias in case selection. In addition, although we collected several external datasets to validate the conclusions obtained in this study, it is still necessary to collect a large amount of clinical case data to further validate the accuracy of the results. Finally, further in vivo and in vitro experiments are needed to validate the specific mechanisms and functions of CDH5, MMP9, MAPK1, and MMP13 in ccRCC vasculogenic mimicry.

## Conclusion

In this study, we constructed a Vasculogenic mimicry related index (VMRI) based on four vasculogenic mimicry related genes (CDH5, MMP9, MAPK1 and MMP13). We found that VMRI can be used as a marker for renal clear cell carcinoma staging and can effectively predict the prognosis and immunotherapy outcome of patients with renal clear cell carcinoma, which was validated by an external dataset. In addition, we identified natural small molecule drugs that can target vasculogenic mimicry-related core target proteins by molecular docking, and finally, we verified that CDH5 promotes the proliferation and invasion of renal clear cell carcinoma by in vitro experiments. In summary, we analyzed the multifaceted comprehensive analysis of renal clear cell carcinoma based on VMRI constructed from vasculogenic mimicry-related genes, and we found that VMRI could effectively predict the prognosis and immunotherapy effect of renal clear cell carcinoma patients and validate it by external datasets. We also identified new prognostic and therapeutic biomarkers for ccRCC as well as targeted small molecule drugs from an vasculogenic mimicry perspective, which provides reliable clues for future precision treatment of renal clear cell carcinoma. In an era when immunotherapy holds great promise for cancer treatment, VMRI provides guiding value for clinical diagnosis and individualized comprehensive treatment of renal clear cell carcinoma.

### Electronic supplementary material

Below is the link to the electronic supplementary material.

Supplementary Material 1

## Data Availability

All data utilized in this study are included in this article and all data supporting the findings of this study are available on reasonable request from the corresponding author.

## Abbreviations

TCGA: The Cancer Genome Atlas
GEO: Gene Expression Omnibus
BCa: Bladder cancer
GTEx: Genotype-Tissue Expression Program
VMRI: Vasculogenic mimicry related index
ROC: Receiver Operating Characteristic
PD-1: Programmed cell death 1
PD-L1: Programmed cell death 1 ligand 1
CTLA4: Cytotoxic T-lymphocyte-associated protein 4
ICB: Immune checkpoint blockade
OS: Overall survival
ROC: Receiver Operation Characteristic
NES: Normalized enrichment score
TMB: Tumor mutation burden
IC50: Half maximal inhibitory concentration
GDSC: Genomics of Drug Sensitivity in Cancer
PPI: Protein–protein interaction
KM: Kaplan–Meier
ssGSEA: Single-sample gene set enrichment analysis
TIDE: Tumor immune dysfunction and exclusion
GSEA: Gene set enrichment analysis
GO: Gene Ontology
KEGG: Kyoto Encyclopedia of Genes and Genomes
LAG3: Lymphocyte Activating 3
PFS: Progression-free survival
DSS: Disease-free survival
TP53: Tumor Protein P53
ACC: Adrenocortical carcinoma
BLCA: Bladder Urothelial Carcinoma, BRCA:Breast invasive carcinoma
CESC: Cervical squamous cell carcinoma and endocervical adenocarcinoma
CHOL: Cholangiocarcinoma
COAD: Colon adenocarcinoma
DLBC: Lymphoid Neoplasm Diffuse Large B-cell Lymphoma
ESCA: Esophageal carcinoma
GBM: Glioblastoma multiforme
HNSC: Head and Neck squamous cell carcinoma
KICH: Kidney Chromophobe
KIRC: Kidney renal clear cell carcinoma
KIRP: Kidney renal papillary cell carcinoma
LAML: Acute Myeloid Leukemia
LGG: Brain Lower Grade Glioma
LIHC: Liver hepatocellular carcinoma
LUAD: Lung adenocarcinoma
LUSC: Lung squamous cell carcinoma
MESO: Mesothelioma
OV: Ovarian serous cystadenocarcinoma
PAAD: Pancreatic adenocarcinoma
PCPG: Pheochromocytoma and Paraganglioma
PRAD: Prostate adenocarcinoma
READ: Rectum adenocarcinoma
SARC: Sarcoma
SKCM: Skin Cutaneous Melanoma
STAD: Stomach adenocarcinoma
TGCT: Testicular Germ Cell Tumors
THCA: Thyroid carcinoma
THYM: Thymoma
UCEC: Uterine Corpus Endometrial Carcinoma
UCS: Uterine Carcinosarcoma
UVM: Uveal Melanoma

## References

[1] Hora M, Albiges L, Bedke J, Campi R, Capitanio U, Giles RH (2023). European Association of Urology Guidelines Panel on Renal Cell Carcinoma Update on the New World Health Organization Classification of Kidney tumours 2022: the urologist’s point of View. Eur Urol.

[2] Moch H, Amin MB, Berney DM, Compérat EM, Gill AJ, Hartmann A (2022). The 2022 World Health Organization Classification of Tumours of the urinary system and male genital organs-Part A: renal, Penile, and testicular tumours. Eur Urol.

[3] Hsieh JJ, Purdue MP, Signoretti S, Swanton C, Albiges L, Schmidinger M (2017). Renal cell carcinoma. Nat Reviews Disease Primers.

[4] Escudier B, Porta C, Schmidinger M, Rioux-Leclercq N, Bex A, Khoo V (2019). Renal cell carcinoma: ESMO Clinical Practice guidelines for diagnosis, treatment and follow-up†. Annals Oncology: Official J Eur Soc Med Oncol.

[5] Borcherding N, Vishwakarma A, Voigt AP, Bellizzi A, Kaplan J, Nepple K (2021). Mapping the immune environment in clear cell renal carcinoma by single-cell genomics. Commun Biology.

[6] Jayson GC, Kerbel R, Ellis LM, Harris AL (2016). Antiangiogenic therapy in oncology: current status and future directions. Lancet (London England).

[7] Thibault C, Fléchon A, Albiges L, Joly C, Barthelemy P, Gross-Goupil M et al. Gemcitabine plus platinum-based chemotherapy in combination with bevacizumab for kidney metastatic collecting duct and medullary carcinomas: Results of a prospective phase II trial (BEVABEL-GETUG/AFU24). European journal of cancer (Oxford, England: 1990). 2023;186:83–90. 10.1016/j.ejca.2023.03.018 37054556

[8] Saliby RM, El Zarif T, Bakouny Z, Shah V, Xie W, Flippot R et al. Circulating and intratumoral immune determinants of response to atezolizumab plus bevacizumab in patients with variant histology or sarcomatoid renal cell carcinoma. Cancer Immunol Res. 2023. 10.1158/2326-6066.CIR-22-0996 PMC10526700 37279009

[9] Ebos JM, Lee CR, Cruz-Munoz W, Bjarnason GA, Christensen JG, Kerbel RS (2009). Accelerated metastasis after short-term treatment with a potent inhibitor of tumor angiogenesis. Cancer Cell.

[10] Herrera-Vargas AK, García-Rodríguez E, Olea-Flores M, Mendoza-Catalán MA, Flores-Alfaro E, Navarro-Tito N (2021). Pro-angiogenic activity and vasculogenic mimicry in the tumor microenvironment by leptin in cancer. Cytokine Growth Factor Rev.

[11] Liu Q, Zhao E, Geng B, Gao S, Yu H, He X (2022). Tumor-associated macrophage-derived exosomes transmitting miR-193a-5p promote the progression of renal cell carcinoma via TIMP2-dependent vasculogenic mimicry. Cell Death Dis.

[12] He M, Yang H, Shi H, Hu Y, Chang C, Liu S (2022). Sunitinib increases the cancer stem cells and vasculogenic mimicry formation via modulating the lncRNA-ECVSR/ERβ/Hif2-α signaling. Cancer Lett.

[13] Wang J, Xia W, Huang Y, Li H, Tang Y, Li Y (2022). A vasculogenic mimicry prognostic signature associated with immune signature in human gastric cancer. Front Immunol.

[14] Xu Y, Sun X, Liu G, Li H, Yu M, Zhu Y (2023). Integration of multi-omics and clinical treatment data reveals bladder cancer therapeutic vulnerability gene combinations and prognostic risks. Front Immunol.

[15] Cannell IG, Sawicka K, Pearsall I, Wild SA, Deighton L, Pearsall SM (2023). FOXC2 promotes vasculogenic mimicry and resistance to anti-angiogenic therapy. Cell Rep.

[16] Huang S, Wang X, Zhu Y, Wang Y, Chen J, Zheng H (2023). SOX2 promotes vasculogenic mimicry by accelerating glycolysis via the lncRNA AC005392.2-GLUT1 axis in colorectal cancer. Cell Death Dis.

[17] Qin LN, Zhang H, Li QQ, Wu T, Cheng SB, Wang KW (2024). Vitamin D binding protein (VDBP) hijacks twist1 to inhibit vasculogenic mimicry in hepatocellular carcinoma. Theranostics.

[18] Zhang J, Bajari R, Andric D, Gerthoffert F, Lepsa A, Nahal-Bose H (2019). Int Cancer Genome Consortium Data Portal Nat Biotechnol.

[19] Rosenberg JE, Hoffman-Censits J, Powles T, van der Heijden MS, Balar AV, Necchi A (2016). Atezolizumab in patients with locally advanced and metastatic urothelial carcinoma who have progressed following treatment with platinum-based chemotherapy: a single-arm, multicentre, phase 2 trial. Lancet (London England).

[20] Uhlén M, Fagerberg L, Hallström BM, Lindskog C, Oksvold P, Mardinoglu A (2015). Proteomics. Tissue-based map of the human proteome.

[21] Yoshihara K, Shahmoradgoli M, Martínez E, Vegesna R, Kim H, Torres-Garcia W (2013). Inferring tumour purity and stromal and immune cell admixture from expression data. Nat Commun.

[22] Jiang P, Gu S, Pan D, Fu J, Sahu A, Hu X (2018). Signatures of T cell dysfunction and exclusion predict cancer immunotherapy response. Nat Med.

[23] Charoentong P, Finotello F, Angelova M, Mayer C, Efremova M, Rieder D (2017). Pan-cancer immunogenomic analyses reveal genotype-immunophenotype relationships and predictors of response to checkpoint blockade. Cell Rep.

[24] Hung SW, Gaetani M, Li Y, Tan Z, Zheng X, Zhang R (2024). Distinct molecular targets of ProEGCG from EGCG and superior inhibition of angiogenesis signaling pathways for treatment of endometriosis. J Pharm Anal.

[25] Cai X, Wang Z, Lin S, Chen H, Bu H (2024). Ginsenoside Rg3 suppresses vasculogenic mimicry by impairing DVL3-maintained stemness via PAAD cell-derived exosomal miR-204 in pancreatic adenocarcinoma. Phytomedicine.

[26] Xu Y, Li Q, Lin H (2024). Bioinformatics analysis of CMTM family in pan-cancer and preliminary exploration of CMTM6 in bladder cancer. Cell Signal.

[27] Huang M, Lin Y, Wang C, Deng L, Chen M, Assaraf YG (2022). New insights into antiangiogenic therapy resistance in cancer: mechanisms and therapeutic aspects. Drug Resist Updates: Reviews Commentaries Antimicrob Anticancer Chemother.

[28] Treps L, Faure S, Clere N (2021). Vasculogenic mimicry, a complex and devious process favoring tumorigenesis - interest in making it a therapeutic target. Pharmacol Ther.

[29] Luo Q, Wang J, Zhao W, Peng Z, Liu X, Li B (2020). Vasculogenic mimicry in carcinogenesis and clinical applications. J Hematol Oncol.

[30] Argilés JM, López-Soriano FJ, Stemmler B, Busquets S (2023). Cancer-associated cachexia - understanding the tumour macroenvironment and microenvironment to improve management. Nat Rev Clin Oncol.

[31] Mao XG, Xue XY, Wang L, Zhang X, Yan M, Tu YY (2013). CDH5 is specifically activated in glioblastoma stemlike cells and contributes to vasculogenic mimicry induced by hypoxia. Neurooncology.

[32] Cai HP, Wang J, Xi SY, Ni XR, Chen YS, Yu YJ (2019). Tenascin-cmediated vasculogenic mimicry formation via regulation of MMP2/MMP9 in glioma. Cell Death Dis.

[33] Lin H, Pan JC, Zhang FM, Huang B, Chen X, Zhuang JT (2015). Matrix metalloproteinase-9 is required for vasculogenic mimicry by clear cell renal carcinoma cells. Urol Oncol.

[34] Li Y, Sun B, Zhao X, Wang X, Zhang D, Gu Q (2017). MMP-2 and MMP-13 affect vasculogenic mimicry formation in large cell lung cancer. J Cell Mol Med.

[35] Braicu C, Buse M, Busuioc C, Drula R, Gulei D, Raduly L et al. A Comprehensive Review on MAPK: a promising therapeutic target in Cancer. Cancers. 2019;11(10). 10.3390/cancers11101618 PMC6827047 31652660

[36] Dhillon AS, Hagan S, Rath O, Kolch W (2007). MAP kinase signalling pathways in cancer. Oncogene.

[37] Zuo GY, Han ZQ, Hao XY, Han J, Li ZS, Wang GC (2014). Synergy of aminoglycoside antibiotics by 3-Benzylchroman derivatives from the Chinese drug Caesalpinia sappan against clinical methicillin-resistant Staphylococcus aureus (MRSA). Phytomedicine: Int J Phytotherapy Phytopharmacology.

[38] Li Y, Ishibashi M, Chen X, Ohizumi Y (2003). Littorachalcone, a new enhancer of NGF-mediated neurite outgrowth, from Verbena Littoralis. Chem Pharm Bull.

[39] Wiatrak B, Gębarowski T, Czwojdziński E, Gąsiorowski K, Tylińska B. Lysosomal Exocytosis of Olivacine on the way to explain Drug Resistance in Cancer cells. Int J Mol Sci. 2022;23(11). 10.3390/ijms23116119 PMC9181543 35682799

