# Supplementary material for: The vasculogenic mimicry related signature predicts the prognosis and immunotherapy response in renal clear cell carcinoma
Source: BMC Cancer. 2024 Apr 5;24:420. doi: 10.1186/s12885-024-12107-x (PMC10996246; doi:10.1186/s12885-024-12107-x)
Supplement: Supplementary file 1 — Supplementary Material 1 [file 12885_2024_12107_MOESM1_ESM.docx]

**Table S1. Vascular mimicry-related genes.**

CDH5

VEGFA

TFPI

SERPINF1

TF

MAPK1

PIK3CA

ROCK1

NOTCH1

ROCK2

MAPK3

EPHA2

LAMC2

KDR

PTGS2

MMP9

SNAI1

TWIST1

MMP2

LOXL2

TFPI2

SNAI2

TGFB1

TWIST2

S1PR1

CD231

CD133

ALDH1

TGFB

HIF1A

LAMC2

MT-CO2

BRCA1

LRIG1

ERBB2

DDAH1

TWIST76

RGS3

LATS2

EPHA2 MMP13 HDAC3

**Table S2. The coefficients of the four genes for the construction of the VMRI.**

id coef

CDH5 -0.0248403025478671

MMP9 0.00786925082279575

MAPK1 -0.0563421696220969

MMP13 0.0558375353173887

**Table S3.** **Links to Web sites with information on small molecule compounds**

Crassifogenin C: https://pubchem.ncbi.nlm.nih.gov/compound/70680261

Episappanol: https://pubchem.ncbi.nlm.nih.gov/compound/13846650

Gnetuhainin D : https://pubchem.ncbi.nlm.nih.gov/compound/10814212

Sappanone B : https://pubchem.ncbi.nlm.nih.gov/compound/13888976

Isolappaol C: https://pubchem.ncbi.nlm.nih.gov/compound/146075482

Leonoside F: https://pubchem.ncbi.nlm.nih.gov/substance/274025462

Licorice Glycoside B: https://pubchem.ncbi.nlm.nih.gov/compound/101938904

Littorachalcone: https://pubchem.ncbi.nlm.nih.gov/compound/10029455

Olivacine: https://pubchem.ncbi.nlm.nih.gov/compound/96364

Picrasidine I: https://pubchem.ncbi.nlm.nih.gov/compound/5324360

Sagecoumarin: https://pubchem.ncbi.nlm.nih.gov/compound/15491283

Thalifaretine: https://pubchem.ncbi.nlm.nih.gov/substance/273393748

Andalasin A: https://pubchem.ncbi.nlm.nih.gov/compound/44576243

Dracunculifoside B: https://pubchem.ncbi.nlm.nih.gov/substance/273208402

Glansreginin B: https://pubchem.ncbi.nlm.nih.gov/substance/318716244

Monocaffeyltartaric Acid: https://pubchem.ncbi.nlm.nih.gov/compound/129716404


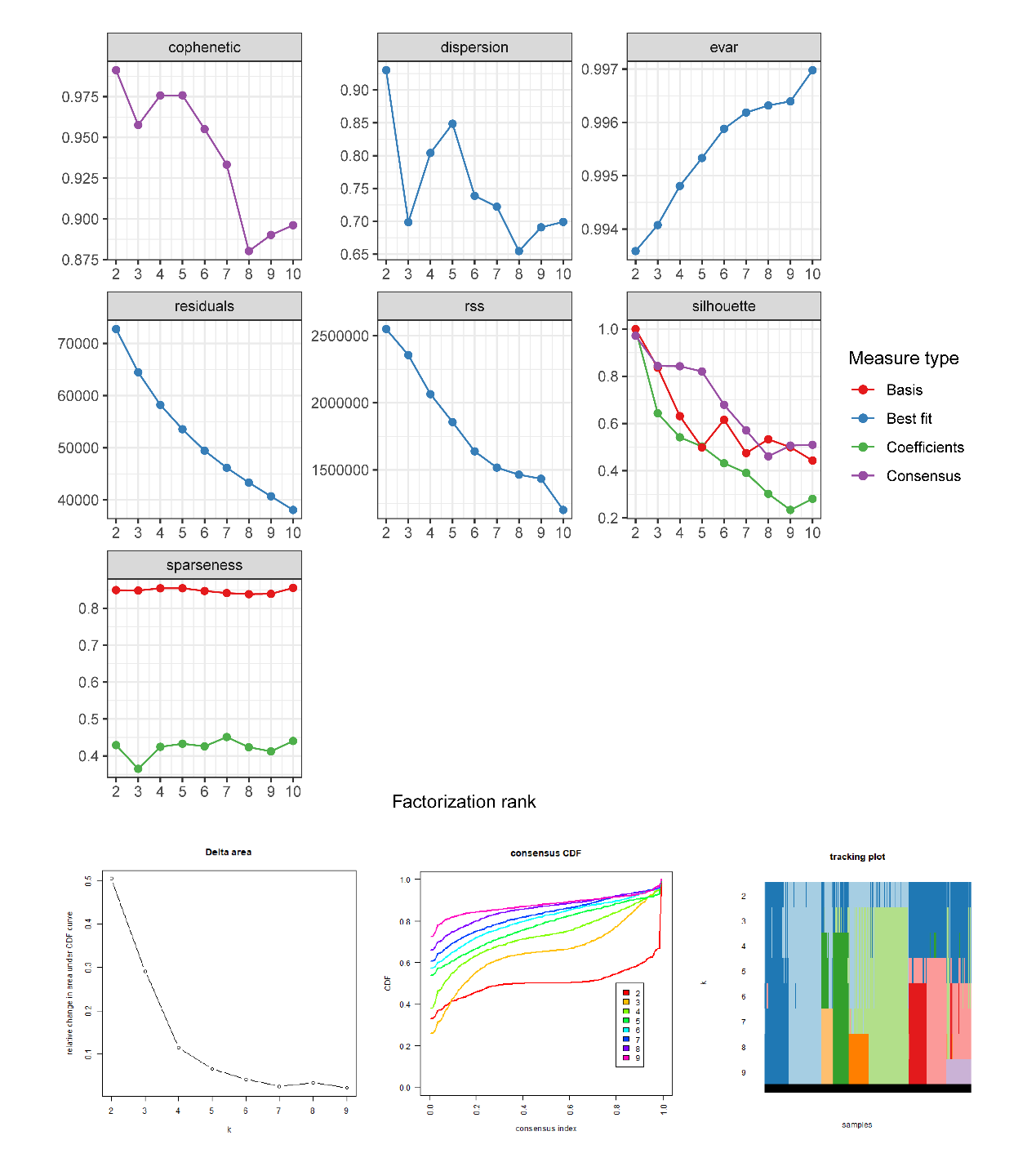


**Figure S1. Cluster analysis.**
